# The Concentrations of Interleukin-6, Insulin, and Glucagon in the Context of Obesity and Type 2 Diabetes and Single Nucleotide Polymorphisms in *IL6* and *INS* Genes

**DOI:** 10.1155/2024/7529779

**Published:** 2024-01-12

**Authors:** Magdalena Król-Kulikowska, Iwona Urbanowicz, Marta Kepinska

**Affiliations:** ^1^Department of Pharmaceutical Biochemistry, Faculty of Pharmacy, Wroclaw Medical University, Wroclaw 50-556, Poland; ^2^Department of Clinical Chemistry and Laboratory Hematology, Faculty of Pharmacy, Wroclaw Medical University, Wroclaw 50-556, Poland

## Abstract

Obesity and diabetes are a problem of modern medicine. Although the environmental factors contributing to the development of these diseases are widely known, research into genetic factors is still ongoing. At the same time, the role of inflammation in the pathophysiology of obesity and diabetes is increasingly emphasized. Therefore, the purpose of this study was to investigate the influence of two selected polymorphisms (rs1800795 and rs3842729) on the development of obesity and type 2 diabetes. In this study, 118 participants were examined, including a control group (nonobese and nondiabetic group), an obese group, and a diabetic group. Genotype analysis was performed using the PCR-RFLP method. It has been shown that in patients with the G/G genotype within the rs1800795 polymorphism *(IL6)*, the chance of developing type 2 diabetes is several times lower compared to patients with the G/C and C/C genotypes. However, the rs3842729 polymorphism *(INS)* does not directly affect the risk of obesity or type 2 diabetes (T2D), although elevated insulin concentrations have been observed in obese and diabetic patients. These results confirm the impact of the rs1800795 polymorphism on the development of diabetes; however, this relationship is more complex and requires further research on other factors.

## 1. Introduction

Obesity is a growing problem in the healthcare system today. It is associated with many other diseases, including insulin resistance and diabetes [1, 2]. The discovery that obesity itself causes inflammation in metabolic tissues initiated a field of research that focuses on the mechanisms of inflammation in obese people [3, 4]. This inflammation is chronic and has a low degree of severity. It is coordinated by metabolic cells in response to excess nutrients and energy [5]. One of the mediators of the inflammatory response, interleukin-6 (IL-6), is strongly associated with inflammation, which is present in both obesity and type 2 diabetes (T2D) [6, 7]. In obesity, adipose tissue immune cells function as the main source of increased levels of circulating IL-6 [8, 9]. The concentration of this cytokine is associated with increased fat mass not only in rodent experimental models but also in obese people [10]. IL-6 is also considered a hormone that participates in immune responses, influencing the metabolism of glucose, proteins, and lipids [11]. This interleukin is also considered to be the initiator of insulin resistance because it has been shown that acute peripheral infusion of IL-6 may impair the action of insulin in mice [12, 13].

The human *IL6* is located at position 7p15–21 and contains five exons and four introns. *IL6* polymorphisms are associated with various diseases, including T2D [14, 15]. One of the most thoroughly investigated single nucleotide polymorphisms (SNPs) found in *IL6,* −174G > C (rs1800795), in the literature, has been frequently associated with a higher risk of developing T2D in different ethnic groups [16, 17]. There are also confirmations that it is linked to the development of obesity [18]. Damavandi et al. [19] constructed genetic models to predict obesity risk, where one of the elements was the rs1800795 polymorphism. Other polymorphisms included in these models are rs4994 *(ADRB3)*, rs9939609 *(FTO)*, rs1042714 *(ADRB2)*, and rs1801133 *(MTHFR)*.

Important peptide hormones associated with obesity and diabetes are glucagon and insulin. Insulin plays an important role in the metabolism of carbohydrates, but also proteins and lipids. Acting together with its opposite hormone, glucagon, it ensures the maintenance of normal glucose levels in the body [20]. Glucose is essential for the functioning of virtually all organs, therefore, disturbances in the synthesis or secretion of insulin may contribute to the development of many diseases, including insulin resistance and diabetes [21]. It has been shown that there is a relationship between the presence of insulin gene *(INS)* polymorphisms and an increased risk of developing many diseases [22–24]. One of them is the −23HphI polymorphism (rs689) found in the 5′untranslated region (5′UTR), which is associated with an increased risk of insulin resistance, type 1 diabetes (T1D), T2D, latent autoimmune diabetes of adults (LADA), hypertension, inflammatory processes, but also colorectal cancer (CRC) [25]. Another polymorphism, 2221MspI (rs3842729) found in the upstream region, is not as well understood as the mentioned rs689 but according to the available literature, it is also associated with the occurrence of T1D [26, 27]. Lewandowski et al. [28] have already conducted studies on similar groups (obese and diabetic groups) in the context of the impact of the rs3842729 polymorphism on the risk of obesity or type 2 diabetes; however, the impact of this polymorphism on these diseases was not confirmed at that time.

The abovementioned examples confirm that *IL6* and *INS* polymorphisms can be associated with different types/subtypes of diabetes, including T2D and other ailments accompanying it. Therefore, the assessment of the effect of the presence of *IL6* and *INS* polymorphisms in patients with obesity or T2D seems to be an interesting and justified endeavour. In addition, even though these diseases are the scourge of the 21st century and numerous studies are carried out on them, still many processes in the human body have not been fully explained. The study aimed to evaluate the impact of selected polymorphisms in the *IL6* (rs1800795) and *INS* (rs3842729) on the development of obesity and T2D, as well as to investigate the relationship between the specific genotypes and plasma glucose levels, serum IL-6 levels, serum insulin levels, serum glucagon levels, and serum C-reactive protein (CRP) levels. In addition, the concentration of copper and zinc in the serum and cadmium in the erythrocyte lysate were also measured, because progress in the development of T2D may also lead to disturbances in the metabolism of trace elements such as zinc, copper, chromium, iron, or cadmium [29]. Moreover, disturbances in the status of trace elements and an increase in oxidative stress may also contribute to insulin resistance and the development of diabetic complications.

## 2. Materials and Methods

### 2.1. Study Groups

Selected SNPs and other parameters were analyzed in a group of 118 people, of whom 23 people had T2D (diabetic group), 45 people had a BMI indicating obesity (obese group), and 50 people constituted the control group. The biological material of patients from the control group and the obese group was obtained as part of the cooperation between the Wrocław Medical University and the Research Network Łukasiewicz-PORT Polish Center for Technology Development. Exclusion criteria for the control group were cardiovascular disease, liver dysfunction (based on the measurement of GGT activity, ALT, and ASP), atherosclerosis, diabetes (based on the measurement of insulin and fasting glucose), hypertension (blood pressure measurement), inflammation (based on the concentration of C-reactive protein), and tumors. Also, the use of drugs or dietary supplements in the last 6 months was used as an exclusion criterion. Obesity was determined based on the WHO BMI cut-off value (30 BMI) [30].

Diabetic patients were recruited in the years 2018-2019 in the nonpublic healthcare center “Krynica” in Wrocław. Type 2 diabetes was confirmed by a physician during recruitment. Additionally, participants completed personal questionnaires in which they answered questions related to their lifestyle, including smoking (Questionnaire S1, Supplementary materials). Furthermore, the concentration of cotinine as a metabolite of nicotine was measured in all respondents. Based on the responses to the personal questionnaire and cotinine concentration, the respondents were divided into smokers (cotinine concentration ≥10 ng/mL) and nonsmokers (cotinine concentration ≤10 ng/mL). The questionnaires also included a question about the drugs used. The vast majority of patients used preparations containing metformin, as well as drugs from the group of angiotensin-converting enzyme inhibitors (ACEi) or rosuvastatin. Only one patient was taking human insulin analogs. Patients were also asked about comorbidities, where the vast majority indicated that they also suffer from hypertension and some of them from hypercholesterolemia. The characteristics of the study groups are presented in Table 1.

The impact of the rs3842729 polymorphism in the context of obesity or type 2 diabetes has already been analyzed in similar groups (obese and diabetic groups) [28, 31]. However, those studies focused mainly on the risk assessment of the abovementioned diseases and not on the concentration of individual parameters such as glucose, insulin, IL-6, glucagon, CRP, zinc, copper, or cadmium in all study groups. The studies showed only a comparison of glucose, insulin, and metal concentrations (zinc, copper, and cadmium) depending on the analyzed genotype within the diabetic group.

### 2.2. Materials

The material for the research was blood samples obtained from the biobank of the Polish Center for Technology Development (Wrocław, Poland) and blood samples obtained from patients diagnosed with T2D. The diagnosis of T2D and the collection of blood from individuals with this disease were performed at the “Krynica” nonpublic healthcare center in Wrocław. Diabetes was diagnosed based on glucose and insulin levels as well as the HOMA-IR index.

Before starting the study, all persons familiarized themselves with the research issues and gave their written consent to have their biological material collected. Before the start of the study, the consent of the Bioethics Committee of the Wroclaw Medical University (no. KB 256/2019) was obtained for the use of the collected biological material for research purposes. To obtain the test material, venous blood was collected in test tubes with clotting activators or an anticoagulant. The content of the clot activator tube (cat. no. 368815, Becton Dickinson, Germany) was used to obtain the serum according to the standard procedure. The content of the tubes with anticoagulants, EDTA (cat. no. 367864, Becton Dickinson, Germany) and gel heparin (cat. no. 368886, Becton Dickinson, Germany), was used for DNA isolation and plasma production.

### 2.3. Methods

#### 2.3.1. Determination of the Concentration of Metals

The concentrations of copper (Cu) and zinc (Zn) in the blood serum and cadmium (Cd) in the whole blood were determined on the SOLAAR M6 atomic absorption spectrophotometer (Thermo Elemental, Solaar House, Cambridge, UK) at the Laboratory of Atomic Absorption Spectrometry, Department and Clinic of Internal Diseases, Vocational, Hypertension, and Clinical Oncology, Wroclaw Medical University. The concentration of Cu and Zn was measured using the flame atomic absorption spectrometry (FAAS) method in an air-acetylene flame, while Cd concentration was measured using the graphite furnace atomic absorption spectrometry (GFAAS) method in a Massmann graphite cuvette.

#### 2.3.2. Determination of Insulin, IL-6, Glucagon, CRP, and Cotinine Concentration

Serum insulin concentration was measured with the Mercodia Insulin ELISA test (cat. no. 10-1113-01, Mercodia AB, Sweden). Serum IL-6 concentration was measured with the Human IL-6 DuoSet ELISA test (cat. no. DY206-05, R&D Systems Europe, Ltd., UK). Serum glucagon concentration was measured with the Human Glucagon ELISA Kit (cat. no. E1266 h, EIAab Science, China). Serum high-sensitivity C-reactive protein (hs-CRP) concentration was measured by the turbidimetric method with the C-reactive protein hs test (cat. no. 31927, Biosystems, Spain). Serum cotinine concentration was measured with the Cotinine ELISA test (cat. no. EIA-3242, DRG International, Springfield, NJ, USA).

#### 2.3.3. Genotyping Analysis

DNA was isolated from the buffy coat using the Syngen Blood/Cell DNA Mini Kit (cat. no. SY221012, Syngen Biotech, Wrocław, Poland). The rs3842729 polymorphism in the *INS* and rs1800795 in the *IL6* was determined using the polymerase chain reaction and restriction fragment length polymorphism analysis (PCR-RFLP). Primers were designed with the Primer-BLAST program based on gene sequences from GenBank (National Center for Biotechnology Information). The sequences of the primers, the conditions of the reactions performed, and the names of the restriction enzymes used are presented in Table 2.

The digested DNA fragments were visualized in 2% agarose gel (cat. no. SY521011, Syngen Biotech, Wrocław, Poland) with Green DNA Gel Stain (cat. no. SY521032, Syngen Biotech, Wrocław, Poland). Exemplary electrophoregrams showing restriction digest products are provided in Figures S1 and S2 (Supplementary materials).

#### 2.3.4. Statistical Analysis

The analyses were carried out using the STATISTICA 13.3 (Statsoft Polska, Sp. z o.o.) package under Wroclaw Medical University's license. The normality of the distribution of variables was checked using the Shapiro–Wilk test and the homogeneity of variance using Levene's test. In order to test statistically significant differences between the two groups, the parametric Student's *t*-test (for variables with a normal distribution) or the nonparametric Mann–Whitney *U* test (for variables that did not meet the conditions of normal distribution) was used. To test statistically significant differences between three or more groups, the nonparametric Kruskal–Wallis test was used (for variables that did not meet the conditions of normal distribution). The differences in frequencies of genotypes were compared using a *χ*^2^ test. The logistic regression analysis was performed to assess the significance of the effect of polymorphism genotypes on the risk of diseases, which was expressed as an odds ratio (OR) at 95% confidence interval (CI). Statistical significance was assumed for *p* < 0.05.

## 3. Results

### 3.1. The Concentrations of IL-6 and Glucagon in the Obesity and the Diabetes Groups

Statistically higher levels of IL-6 were observed in both obese (*p*=0.022) and diabetic (*p* < 0.001) groups compared to the control group. In turn, higher levels of glucagon were observed in the diabetic group compared to the control (*p* < 0.000) and obese (*p*=0.001) groups. The obtained results are presented in Table 3. Concentrations of glucose, insulin, CRP, and selected metals (zinc, copper, and cadmium) between these groups have already been presented in previous studies [28, 31].

### 3.2. The Influence of the rs1800795 Polymorphism in IL6 on the Concentrations of the Selected Parameters Associated with Glucose Metabolism and Concentrations of Selected Metals

A relationship between the occurrence of a specific genotype (rs1800795) and the control, the obese, or the diabetic group was noticed (*χ*^2^ = 10.128 and *p* = 0.038). In the control group and the obese group, the percentage of individual genotypes was similar, and the G/G genotype was dominant in them. Meanwhile, in the diabetic group, patients with the G/G genotype constituted only 13.04%. The results are shown in Table 4.

A statistically higher insulin concentration was demonstrated in the obese group with the G/G genotype (rs1800795) compared to the control group with the same genotype (*p* = 0.026) (Table 5). It was similar in the group of patients with the G/C genotype, in which higher insulin concentrations were also shown in the obese group (*p* = 0.004) and diabetic group (*p* = 0.007) compared to the control group. In turn, in the group of patients with the C/C genotype, higher insulin concentration was demonstrated in the diabetic group compared to the control group (*p* = 0.008). However, no significant differences in glucagon concentrations were observed. Only the glucagon concentration in diabetics with the C/C genotype compared to the control group was on borderline statistical significance (*p* = 0.0999).

A statistically lower concentration of IL-6 was observed in obese people with the G/G genotype compared to the control group with the same genotype (*p*=0.014). A lower concentration of IL-6 was also shown in the diabetic group with the C/C genotype compared to the control group (*p*=0.009) and the obese group (*p*=0.044) with the same genotype.

As far as glucose is concerned, the following changes were observed: higher concentration in the diabetic group with the G/G genotype compared to the control group with the same genotype (*p*=0.005); higher concentration in the diabetic group with the G/C genotype compared to the control group (*p* < 0.001) and the obese group (*p*=0.003) with the same genotype; higher concentration in the diabetic group with the C/C genotype compared to the control group with the same genotype (*p* < 0.001).

Higher CRP concentrations were observed in obese patients with the G/G genotype compared to the control group with the same genotype (*p*=0.003). Similarly, higher concentrations of this parameter were noticed in obese patients (*p*=0.006) and diabetic patients (*p*=0.001) with the G/C genotype compared to the control group with the same genotype. Higher CRP concentrations were also observed in obese patients with the C/C genotype compared to patients from the control group with the same genotype (*p*=0.008).

Finally, higher copper concentrations were demonstrated in diabetics with the G/C genotype (*p*=0.002) and the C/C genotype (*p*=0.043) compared to the control groups with the corresponding genotypes. In the case of cadmium, a higher concentration of this element was observed in the group of diabetics with the G/G genotype compared to the control group with the same genotype (*p*=0.019) and the obese group with the same genotype (*p*=0.017). Moreover, a higher concentration of cadmium was found in the group of diabetics with the G/C genotype compared to the control group (*p*=0.013), as well as a lower concentration of this element in the same group compared to obese patients (*p* < 0.001). A similar relationship was also observed in the case of the C/C genotype (*p*=0.026 and *p*=0.015, respectively). No significant differences in zinc concentrations were observed. All of the above results are shown in Table 5.

### 3.3. The Influence of the rs3842729 Polymorphism in INS on the Concentrations of the Selected Parameters Associated with Glucose Metabolism and Concentrations of Selected Metals

There was no statistically significant difference in the genotypic distribution (rs3842729) between the control group, the group of obese patients, and the group of diabetic patients (*χ*^2^ = 5.033 and *p* = 0.081). The results are shown in Table 6.

**Figure 1:**
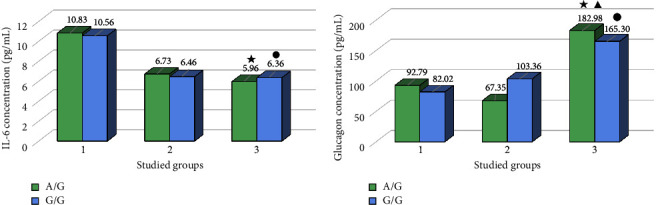
(a) The IL-6 concentration in studied groups in terms of rs3842729 polymorphism *(INS)*. (b) The glucagon concentration in studied groups in terms of rs3842729 polymorphism *(INS)*. 1: control group, 2: obese group, 3: diabetic group, A/G: patients with the A/G genotype (rs3842729 polymorphism), and G/G: patients with the G/G genotype (rs3842729 polymorphism). The asterisk marks a statistically significant difference compared with the control group with A/G genotype (*p* < 0.05). The circle marks a statistically significant difference compared with the control group with G/G genotype (*p* < 0.05). The triangle marks statistically significant difference compared with the obese group with A/G genotype (*p* < 0.05).

A statistically lower IL-6 concentration was found in diabetic patients with the A/G genotype (*p*=0.002) and the G/G genotype (*p*=0.025) compared to the control group with corresponding genotypes (Figure 1(a)).

Higher levels of glucagon were observed in the group of diabetics with the A/G genotype compared to the control group (*p*=0.048) with the same genotype, and lower levels of this parameter was noticed in the diabetic group with the A/G genotype compared to the obese group (*p*=0.043) with the same genotype. Moreover, higher levels of glucagon was observed in the group of diabetic patients with the G/G genotype (*p*=0.005) compared to the control group with the same genotype too. These results are shown in Figure 1.

Statistically higher levels of insulin were observed in obese patients with the G/G genotype (*p*=0.003) compared to the control group with the same genotype. Furthermore, higher glucose concentrations were also shown in obese patients with the A/G genotype (*p*=0.015) compared to the control group with the same genotype.

Higher CRP concentrations were observed in obese patients with the A/G genotype (*p* < 0.001) and diabetic patients with the A/G genotype (*p*=0.030) compared to the control group with the same genotype. The situation was similar in the case of patients with the G/G genotype (*p*=0.001 and *p* < 0.001, respectively).

No significant differences were observed for the concentrations of copper and zinc. However, higher levels of cadmium in the obese group with G/G genotype (*p*=0.023) compared to the control group were noticed. All of the above results are shown in Table 7. The concentrations of insulin, glucose, and metals in the group of diabetic patients separated into genotypes (A/G and G/G) in terms of rs3842729 polymorphism in the INS were presented in an earlier publication [28].

### 3.4. The Influence of IL6 and INS Polymorphisms on the Risk of Occurrence of Obesity or Diabetes

This study also looked at the effects of several variables, including genotypic variability, on the development of obesity and T2D using logistic regression. In the context of the risk of obesity development, the following variables were significant: age and smoking. A one-year increase in age has been shown to be associated with an 8.70% (*p* < 0.001) increase in obesity risk. Furthermore, the odds of developing obesity are approximately 3.73-fold (*p*=0.010) higher in cigarette smokers than in people who are not exposed to tobacco smoke.

In turn, in the context of the development of T2D, variables such as age, BMI values, and genotypic variability within the rs1800795 polymorphism have been found to be significant. It has been shown that patients with the G/G genotype are at approximately 4.72-fold (*p*=0.037) lower odds to develop T2D compared to patients with other (G/C and C/C) genotypes. Moreover, it has been noticed that an increase in BMI by one unit increases the chance of developing T2D by approximately 67.20% (*p* < 0.000). As mentioned earlier, the risk also increases with age, but since elderly people predominated in the diabetic population in this study, the OR value may be overestimated, so it was not included. The described results are shown in Tables 8 and 9.

## 4. Discussion

The mechanisms underlying individual differences in obesity predisposition remain unclear. Obesity itself, in turn, may be one of the etiological factors in the development of T2D. The latter involves an insufficient response of pancreatic islet cells to the progressive insulin resistance that is usually associated with aging, low physical activity, and weight gain [32]. The increase in the global prevalence of obesity is related to the growing incidence of T2D (because obesity is one of the etiological factors of T2D), which means that these two have become civilization diseases and serious problems of modern society [33]. Attention should also be paid to the role of impaired response to insulin produced by the pancreas. This phenomenon may have its basis in genetic factors. Differences in the symptoms and course of these diseases in individual patients may result from the participation of many genes and the level of their expression, but also from post-translational changes in the products of these genes. Having a parent with T2D has been shown to increase the risk of developing diabetes. However, the genes whose mutations or structural changes would determine this have not yet been clearly identified [34]. In the development of obesity and T2D, adiponectin should also be taken into account. It significantly affects glucose metabolism as well as tissue sensitivity to insulin, acting as a protective factor in the development of diabetes complications [35]. Tschritter et al. [36] demonstrated a correlation linking three *ADIPOQ* variants (rs17300539, rs3774261, and rs6444175) with insulin sensitivity. Additionally, obese people showed lower adiponectin concentrations compared to the control group.

The influence of genetic polymorphisms, including *IL6* polymorphisms, on the increased risk of obesity and T2D was investigated. Based on logistic regression, it can be concluded that the rs1800795 polymorphism *(IL6)* is a useful prognostic tool in estimating the risk of T2D. The G/G genotype was associated with a 5.75-fold lower risk of T2D compared to the other genotypes. Although in some studies it was the G allele that was associated with an increased risk of T2D development [37, 38], some data indicate that the rs1800795 polymorphism is not associated with the effect on this disease [39, 40]. Moreover, some researchers have obtained opposite results that indicate that the presence of the C allele causes the development of diabetes [16]. These differences usually resulted from the ethnic diversity of the investigated populations. Perhaps interactions with other gene variants or gene-environment interactions are responsible for the interethnic variation. In the case of the second variable, BMI, studies seem to confirm the results of this study indicating that an increase in this parameter increases the chance of developing T2D [41, 42]. The same applies to the risk of obesity, where the present authors have shown that older age and smoking increase the risk of this disease, which has also been confirmed by other publications [43–45].

IL-6 is a mediator of inflammation, therefore, its increased concentration should be observed in diseases related to inflammation [46]. However, in this study, the results showed a decreased concentration in both study groups (obese and diabetic individuals) compared to the control group, which is in contrast to other studies [47]. Derosa et al. [48] showed an increase in the concentration of IL-6 and other inflammatory adipocytokines in the group of obese patients compared to the nonobese group. Perhaps the reason for the discrepancy between the results of this study and the research of other scientists is a too small population of study groups as in the case of Al-Shukaili et al. [49], because the presented study had only 45 participants in the group of obese patients and only 23 in the group of individuals with diabetes. The reason why people from the control group had higher concentrations of IL-6 may also be that the lysate of erythrocytes from this group showed a statistically significant higher concentration of cadmium compared to the diabetic group, which would indicate that people in this group accumulate cadmium which has a proinflammatory effect. Ołdakowska et al. [50] showed a 1.5-fold increase in IL-6 concentration in smokers compared to nonsmokers in the group of patients with acute pancreatitis. In addition, there were more smokers in the control group and in the obese group than in the diabetic group.

The inflammatory reaction is also accompanied by an increase in CRP, which has long been used to assess the clinical condition of patients [51]. It is known that obesity can cause inflammation in metabolic tissues [52]. The presented study showed a higher concentration of CRP in the serum of obese and diabetic patients compared to the control group. The obtained results of studies in the scope of CRP concentration confirm the results of previous reports that obesity and diabetes cause inflammation [52, 53]. However, the results obtained in this study seem to indicate that the presence of the analyzed polymorphisms does not affect the concentration of CRP.

This study also observed the impact of obesity and diabetes on the concentrations of selected elements (copper, zinc, and cadmium). The results regarding this last element seem to be particularly interesting here. Although the studied polymorphisms seem to have no effect on cadmium concentrations, it was observed that both obese and diabetic patients have much higher values compared to the control group. In turn, cadmium, through a number of interactions, adversely affects the pathophysiology of adipose tissue, thus contributing to the increase in insulin resistance and the development of diabetes [54]. The basic mechanism of the toxic effect of cadmium on cells of many tissues and organs is the induction of oxidative stress and peroxidation of lipids included in cell membranes. Under the influence of cadmium, the concentration of ROS increases. The cytotoxicity of this element is limited by a number of antioxidants, such as catalase, superoxide dismutase, or mannitol. In addition, cadmium through the so-called “ion mimicry” can be absorbed by transporters specific for physiologically essential ions, such as iron (Fe), calcium (Ca), or zinc (Zn) [55]. Due to these similarities, the Cd ion competes with the Zn ion for binding to proteins, and thus may displace zinc from its role in the synthesis and “processing of insulin” [56]. The results obtained in this research may also be attributable to the fact that among the obese group, there were more smokers compared to the control group, and the concentration of cadmium is increasing in tobacco smokers [57].

Of particular importance in the development of T2D or obesity may be polymorphisms of the *INS*. Researchers were interested in one of the *INS* polymorphisms, rs689, which was assessed over the years in various populations and different countries. It has been associated with such disease states as T1D, T2D, latent autoimmune diabetes in adults (LADA), obesity, insulin resistance, or hypertension [58–60]. However, few reports have been published so far assessing the relationship between the rs3842729 polymorphism of the *INS* with the development of obesity and T2D. Most publications show the influence of the rs3842729 polymorphism on the incidence of T1D [46, 58]. Lewandowski et al. [31] pointed to the relationship between G/G genotype presence (rs3842729 polymorphism) and an approximately 2.8-fold higher risk of T2D development. In other studies by Lewandowski et al. [28], the influence of this polymorphism on the concentrations of selected parameters in the obese and control groups was not included, which is why this paper presents these results. However, no statistically significant difference in insulin concentration was observed between the genotypes within the rs3842729 polymorphism. In turn, statistically significant higher levels of insulin were found in the serum of obese patients compared to the control group. The results obtained correspond with previous research results which show that the concentration of insulin in obese people may be higher than in people with normal body weight [47].

## 5. Conclusions

The concentrations of the studied parameters (insulin, glucagon, IL-6, glucose, CRP, and selected elements) changed depending on the occurrence of obesity and/or T2D compared to the control group, but this indicates the influence of these diseases and not the studied polymorphisms (rs1800795 and rs3842729).

However, in the case of the rs1800795 polymorphism *(IL6)*, this study demonstrated its effect on T2D. The G/G genotype was associated with a lower risk of T2D compared to the other genotypes (G/C and C/C). However, this result may be biased, because the G/G genotype was present in only 13.04% of diabetic patients (for comparison, the G/C genotype was present in 39.13% of diabetics and the C/C genotype in 47.83% of patients with diabetes). Nevertheless, this result seems promising and may indicate the protective role of the G/G genotype in the context of T2D development. In turn, the rs3842729 polymorphism of the *INS* does not directly affect the risk of obesity or T2D.

In addition, this study found that both age and smoking increase the risk of developing obesity, and confirmed that age and BMI influence the development of T2D. In conclusion, the relationship between the investigated polymorphisms (rs1800795 and rs3842729) and the risk of developing obesity or diabetes is more complex and also results from other factors. Nonetheless, there are some limitations to this study that should be taken into account when drawing conclusions.

One of the main limitations of this study is that the results obtained are based on a population that is not homogeneous. The lack of homogeneity concerns not only the characteristics desirable for the selection of participants (obesity or T2D) but also the heterogeneous ratio of women to men and age in the study groups. This is due, among others, to the specificity of T2D patients, who are usually elderly. To eliminate differences related to sex, additional subgroups (women and men) could be distinguished; however, due to the small size of the study population, this step was abandoned. The small size of the population is another limitation of this study. There are also differences in the number of people between groups.

For this reason, the authors of this article recommend a cautious approach to the conclusions drawn in this study. It should therefore be considered a preliminary study and its results should be validated by wider studies covering a larger population. The next step should be to repeat similar analyses taking into account larger sizes of the relevant study groups. This would help eliminate the uncertainties associated with the results obtained so far.

## Figures and Tables

**Table 1:** The characteristics of the studied groups.

Parameter	Control group (*N* = 50)	Obese group (*N* = 45)	Diabetic group (*N* = 23)
Age (years)	35.76 ± 11.17	48.29 ± 12.93	68.83 ± 7.67

Sex	Men: 21 Women: 29	Men: 24 Women: 21	Men: 6 Women: 17

BMI (kg/m^2^)	23.83 ± 3.37	32.85 ± 2.09	31.28 ± 5.16

Smoking status	Yes: 7 No: 43	Yes: 17 No: 28	Yes: 3 No: 20

HOMA-IR	1.79 ± 1.21	3.59 ± 2.55	6.19 ± 4.79

HbA1c (%)	—	—	6.81 ± 1.27

N: number of patients; HOMA-IR: homeostasis model assessment for insulin resistance; HbA1c: glycated hemoglobin.

**Table 2:** The conditions for PCR and restriction enzyme digestion.

SNP	Primers	PCR-RFLP conditions
rs3842729	Forward primer-5′ GGC TTG ACC GGC CAG GGT GTC CCC 3′ Reverse primer-5′ ACC CCC AGC TGC AAC CTC AGG GGC T 3′	The initial denaturation-95°C for 5 min, denaturation-95°C for 40 s, annealing-67.4°C for 35 s, elongation-72°C for 25 s, the final elongation72°C for 10 min

rs1800795	Forward primer-5′ TGA CTT CAG CTT TAC TCT TTG T 3′ Reverse primer-5′ CTG ATT GGA AAC CTT ATT AG 3′	The initial denaturation-95°C for 15 min, denaturation-95°C for 40 s, annealing-55°C for 35 s, elongation-72°C for 40 s, the final elongation-72°C for 10 min

SNP	Restriction enzymes	Restriction enzyme digestion conditions

rs3842729	MspI	37°C for 1 hour

rs1800795	SfaNI	37°C for 16 hours

**Table 3:** Concentrations of IL-6 and glucagon in the control, obese, and diabetic groups.

Parameters	Control group (*N* = 50)	Obese group (*N* = 45)	Diabetic group (*N* = 23)
IL-6 (pg/mL)	{7.37; **10.61**; 34.30}	{6.22; **6.62**; 9.53}^*∗*^	{5.91; **6.2**1; 7.17}^*∗*^
Glucagon (pg/mL)	{58.41; **86.54**; 120.72}	{44.35; **77.86**; 112.20}	{130.80; **173.18**; 219.27}^*∗*,*∗∗*^

Values shown as {1st quartile; **median**; 3rd quartile}. Data were obtained with the Kruskal–Wallis test with the post hoc Dunn's test. *N*: number of patients; ^*∗*^*p* < 0.05 compared to control group; ^*∗∗*^*p* < 0.05 compared to obese group. Bold values represent medians.

**Table 4:** The genotypic distribution of the rs1800795 polymorphism *(IL6) *in the control, obese, and diabetic groups.

Genotype	Control group *N* = 50	Obese group *N* = 45	Diabetic group *N* = 23
G/G *N* = 36 (30.51%)	*N* = 18 (36.00%)	*N* = 15 (33.33%)	*N* = 3 (13.04%)
G/C *N* = 50 (42.37%)	*N* = 18 (36.00%)	*N* = 23 (51.11%)	*N* = 9 (39.13%)
C/C *N* = 32 (27.12%)	*N* = 14 (28.00%)	*N* = 7 (15.56%)	*N* = 11 (47.83%)

*N*: number of patients. Data were obtained with the chi square test.

**Table 5:** Concentrations of the selected parameters associated with glucose metabolism in terms of rs1800795 *(IL6)*.

Parameter	Control group (*N* = 50)			Obese group (*N* = 45)			Diabetic group (*N* = 23)		
	G/G (*N* = 18)	G/C (*N* = 18)	C/C (*N* = 14)	G/G (*N* = 15)	G/C (*N* = 23)	C/C (*N* = 7)	G/G (*N* = 3)	G/C (*N* = 9)	C/C (*N* = 11)
Insulin (mU/L)	6.40; **7.90**; 9.00	4.20; **6.30**; 8.20	4.50; **5.20**; 6.90	7.90; **13.60**; 14.80^*∗*^	9.65; **14.55**; 17.75^*∗∗∗*^	7.40; **10.25**; 19.30	4.09; **13.92**; 41.70	10.65; **17.39**; 23.17^*∗∗∗*^	8.13; **9.91**; 20.45^$$^
Glucagon (pg/mL)	59.27; **88.35**; 125.93	58.41; **86.54**; 97.54	39.20; **74.36**; 131.80	44.35; **103.36**; 112.20	46.22; **67.35**; 106.85	—	82.88; **170.00**; 299.41	130.80; **173.18**; 195.75	140.13; **181.69**; 221.05
IL-6 (pg/mL)	8.85; **12.02**; 23.68	6.07; **8.90**; 76.92	7.46; **9.91**; 20.31	6.10; **6.40**; 8.78^*∗*^	6.22; **6.87**; 13.52	7.06; **8.74**; 9.90	6.24; **7.17**; 7.18	5.84; **6.01**; 8.94	5.92; **6.08**; 6.29^$$,$$$^
Glucose (mmol/L)	4.56; **4.72**; 4.83	4.44; **4.81**; 5.00	4.44; **4.72**; 5.06	4.72; **5.17**; 5.50	4.92; **5.17**; 5.53	5.03; **5.36**; 6.25	6.04; **9.20**; 11.10^*∗*^	6.60; **6.80**; 9.50^*∗∗∗*^^,$^	6.30; **8.25**; 8.50^$$^
CRP (mg/L)	0.20; **0.47**; 0.80	0.37; **0.89**; 1.15	0.32; **0.61**; 0.91	1.11; **1.22**; 1.48^*∗*^	0.97; **1.34**; 3.19^*∗∗∗*^	1.80; **4.52**; 6.93^$$^	0.62; **1.33**; 14.24	1.38; **2.79**; 5.41^*∗∗∗*^	0.69; **1.04**; 2.25
Cu (*μ*g/L)	960.21; **1012.89**; 1257.32	853.30; **988.99**; 1055.65	912.33; **994.74**; 1059.29	889.84; **1001.59**; 1114.89	991.47; **1075.33**; 1167.87	974.55; **1148.39**; 1321.91	1011.00; **1206.00**; 1490.00	1121.00; **1248.00**; 1325.00^*∗∗∗*^	1016.00; **1082.00**; 1221.00^$$^
Zn (*μ*g/L)	940.35 ± 130.09	969.25 ± 126.96	987.86 ± 151.71	943.94 ± 118.78	985.36 ± 92.38	880.19 ± 133.20	802.00 ± 47.29	902.67 ± 77.86	899.36 ± 102.83
Cd (mg/g Hg)	1.07; **2.46**; 3.39	1.63; **1.87**; 3.56	1.62; **2.43**; 3.64	1.54; **2.91**; 6.04	1.78; **4.22**; 5.50	2.07; **4.49**; 11.13	1.69; **3.09**; 5.15^*∗*^^,∗∗^	2.52; **3.59**; 7.82^*∗∗∗*^^,$^	1.79; **3.83**; 5.04^$$,$$$^

Values are shown as mean value ± standard deviation or {1st quartile; **median**; 3rd quartile}. Data were obtained with the Kruskal–Wallis test with the post hoc Dunn's test. In the case of zinc, data were obtained with the ANOVA. *N*: number of patients; ^*∗*^*p* < 0.05 compared to control group with G/G genotype; ^*∗∗*^*p* < 0.05 compared to obese group with G/G genotype; ^*∗∗∗*^*p* < 0.05 compared to control group with G/C genotype; ^$^*p* < 0.05 compared to obese group with G/C genotype; ^$$^*p* < 0.05 compared to control group with C/C genotype; ^$$$^*p* < 0.05 compared to obese group with C/C genotype. Bold values represent medians.

**Table 6:** The genotypic distribution of the rs3842729 polymorphism *(INS)* in the control, obese, and diabetic groups.

Genotype	Control group *N* = 50	Obese group *N* = 45	Diabetic group *N* = 23
A/G *N* = 61 (51.69%)	*N* = 23 (46.00%)	*N* = 29 (64.44%)	*N* = 9 (39.13%)
G/G *N* = 57 (48.31%)	*N* = 27 (54.00%)	*N* = 16 (35.56%)	*N* = 14 (60.87%)

*N*: number of patients. Data were obtained with the chi square test.

**Table 7:** The concentrations of the selected parameters associated with glucose metabolism in terms of rs3842729 *(INS)*.

Parameters	Control group (*N* = 50)		Obese group (*N* = 45)		Diabetic group (*N* = 23)	
	A/G (*N* = 23)	G/G (*N* = 27)	A/G (*N* = 29)	G/G (*N* = 16)	A/G (*N* = 9)	G/G (*N* = 14)
Insulin (mU/L)	4.90; **7.00**; 8.20	4.70; **6.80**; 9.40	8.90; **10.95**; 17.50^*∗*^	11.50; **14.60**; 18.80^*∗∗∗*^	—	—
Glucagon (pg/mL)	60.90; **92.79**; 120.72	50.92; **82.02**; 119.29	44.35; **67.35**; 112.20	47.60; **103.36**; 106.85	130.80; **182.98**; 221.05^*∗*^^,∗∗^	140.13; **165.30**; 195.75^*∗∗∗*^
IL-6 (pg/mL)	7.99; **10;83**; 30.59	7.28; **10.56**; 38.01	6.28; **6.73**; 11.20	6.22; **6.46**; 9.51	5.82; **5.96**; 6.71^*∗*^	5.98; **6.36**; 7.18^*∗∗∗*^
Glucose (mmol/L)	4.56; **4.78**; 4.94	4.44; **4.64**; 5.06	4.92; **5.17**; 5.56^*∗*^	4.72; **5.17**; 5.61	—	—
CRP (mg/L)	0.37; **0.65**; 0.93	0.19; **0.43**; 0.94	0.99; **1.33**; 2.82^*∗*^	1.14; **1.80**; 5.26^*∗∗∗*^	0.69; **1.33**; 4.07^*∗*^	1.03; **1.72**; 3.79^*∗∗∗*^
Cu (*μ*g/L)	1018.49 ± 114.23	1007.20 ± 200.71	1084.97 ± 136.46	1072.88 ± 173.16	—	—
Zn (*μ*g/L)	924.60 ± 115.18	997.67 ± 141.60	953.02 ± 89.37	953.64 ± 145.95	—	—
Cd (mg/g Hg)	1.49; **2.25**; 3.51	1.46; **2.44**; 3.60	1.59; **2.91**; 4.92	2.73; **6.04**; 14.52^*∗∗∗*^	—	—

Values are shown as mean value ± standard deviation or {1st quartile; **median**; 3rd quartile}. Data were obtained with the Kruskal–Wallis test with the post hoc Dunn's test (glucagon, IL-6, and CRP), with the Mann–Whitney *U* test (insulin, glucose, and cadmium), and with the Student's *t*-test (copper and zinc). *N*: number of patients; ^*∗*^*p* < 0.05 compared to control group with A/G genotype; ^*∗∗*^*p* < 0.05 compared to obese group with A/G genotype; ^*∗∗∗*^*p* < 0.05 compared to control group with G/G genotype. Bold values represent medians.

**Table 8:** Relationship between the selected parameters and the risk of developing obesity.

SNP (*gene*)	Genotype	Obese group	Control group	*p*	OR	95% CI OR
rs1800795 (*IL6*)	G/G	17	18	0.379	1.667	0.535–5.196
	G/C	26	18	0.094	2.556	0.853–7.655
	C/C	14	14	—	1.000	—
rs3842729 (*INS*)	A/G	32	23	—	1.000	—
	G/G	25	27	0.073	0.470	0.206–1.073

Other variables	Category	Obese group	Control group	*p*	OR	95% CI OR

Age (years)	—	—	—	**<0.000**	**1.087**	**1.045–1.131**
Sex	Men	27	21	—	1.000	—
	Women	30	29	0.270	0.634	0.281–1.426
Smoking status	Yes	19	7	**0.010**	**3.730**	**1.371–10.145**
	No	38	43	—	1.000	—

Data were obtained with the logistic regression analysis. OR: odds ratio; CI: confidence interval; statistical significance: *p* < 0.05 Values that are statistically significant are in bold.

**Table 9:** Relationship between the selected parameters and the risk of developing type 2 diabetes.

SNP (*gene*)	Genotype	Diabetic group	Control group	*p*	OR	95% CI OR
rs1800795 (*IL6*)	G/G	3	18	**0.037**	**0.212**	**0.049–0.909**
	G/C	9	18	0.431	0.636	0.207–1.959
	C/C	11	14	—	1.000	—
rs3842729 (*INS*)	A/G	9	23	—	1.000	—
	G/G	14	27	0.583	1.325	0.485–3.621

Other variables	Category	Diabetic group	Control group	*p*	OR	95% CI OR

BMI	—	—	—	**<0.000**	**1.672**	**1.305–2.143**
Sex	Men	6	21	—	1.000	—
	Women	17	29	0.195	2.052	0.692–6.084
Smoking status	Yes	3	7	0.912	0.921	0.216–3.939
	No	20	43	—	1.000	—

Data were obtained with the logistic regression analysis. OR: odds ratio; CI: confidence interval; statistical significance: *p* < 0.05. Values that are statistically significant are in bold.

## Data Availability

The datasets used and/or analyzed during the current study are available from the corresponding author on reasonable request.

## Supplementary Materials

Supplementary Materials The authors provide Supplementary Materials which contain a questionnaire completed by patients with type 2 diabetes and exemplary electrophoregrams for the tested polymorphisms: Questionnaire S1: sample of a questionnaire conducted among people suffering from type 2 diabetes. Figure S1: example of electropherogram for rs3842729 *(INS)*. Figure S2: example of electropherogram for rs1800795 *(IL6)*.
