# Supplementary material for: The Concentrations of Interleukin-6, Insulin, and Glucagon in the Context of Obesity and Type 2 Diabetes and Single Nucleotide Polymorphisms in IL6 and INS Genes
Source: J Obes. 2024 Jan 12;2024:7529779. doi: 10.1155/2024/7529779 (PMC10798838; doi:10.1155/2024/7529779)
Supplement: Supplementary Materials — The authors provide Supplementary Materials which contain a questionnaire completed by patients with type 2 diabetes and exemplary electrophoregrams for the tested polymorphisms: Questionnaire S1: sample of a questionnaire conducted among people suffering from type 2 diabetes. Figure S1: example of electropherogram for rs3842729 (INS). Figure S2: example of electropherogram for rs1800795 (IL6). [file 7529779.f1.docx]

**Supplementary materials**

Questionnaire S1: Sample of a questionnaire conducted among people suffering from type 2 diabetes.

**PART 1. PRELIMINARY INFORMATION**

| Sex  (W/M) | Country | Voivodeship  (birth) | City  of residence | Age | Height | Weight |
| --- | --- | --- | --- | --- | --- | --- |
|  |  |  |  |  |  |  |

Do you suffer from diabetes? If so, what type?

|  |
| --- |

Do you suffer from chronic diseases other than diabetes? If so, which ones?

|  |
| --- |

**PART 2. LIFESTYLE**

| Do you play sports? How many hours per week? |  |
| --- | --- |
| Do you smoke cigarettes? How many years? How many packs a day? |  |
| Do you take medications? Which medications? |  |
| Do you consume alcohol? In what amount (in grams of alcohol)? How often? |  |

**PART 3. DISEASES IN THE FAMILY**

| Do your parents have diabetes? |  |
| --- | --- |
| If so, what type/at what age was it diagnosed? |  |
| Do your siblings have diabetes? |  |
| If so, what type/at what age was it diagnosed? |  |
| Does the father/brother below 55 years of age had a myocardial infarction? |  |
| Does the father/brother below 55 years of age sudden death occurred? |  |
| Does the father/brother below 55 years of age suffered a stroke? |  |
| Does the mother/sister below 65 years of age had a myocardial infarction? |  |
| Does the mother/sister below 65 years of age sudden death occurred? |  |
| Does the mother/sister below 65 years of age suffered a stroke? |  |
| Have your parents been diagnosed with cancer? |  |
| If so, what/at what age? |  |
| Have your siblings been diagnosed with cancer? |  |
| If so, what/at what age? |  |


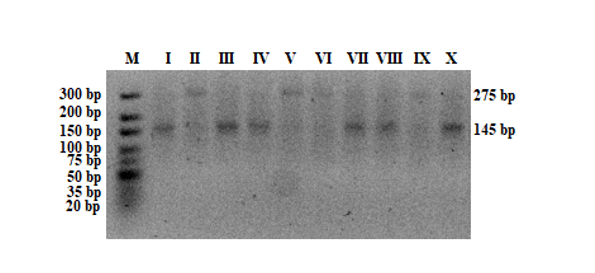


Figure S1: Example of electropherogram for rs3842729 (*INS*).

M – marker ladder; II, V, VI, IX – A/G genotype; I, III, IV, VII, VIII, X – G/G genotype.


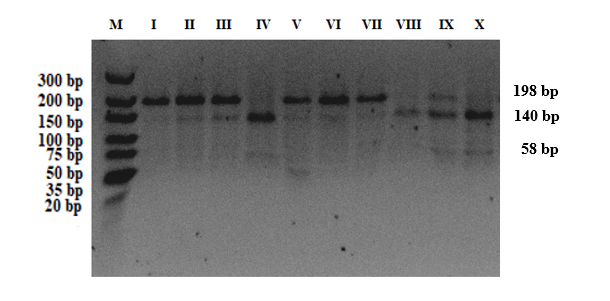


Figure S2: Example of electropherogram for rs1800795 (*IL6*).

M – marker ladder; I – G/G genotype; II, III, V, VI, VII, IX – G/C genotype; IV, VIII, X – C/C genotype.
